# Identification of Residues Involved in Substrate Specificity and Cytotoxicity of Two Closely Related Cutinases from *Mycobacterium tuberculosis*

**DOI:** 10.1371/journal.pone.0066913

**Published:** 2013-07-02

**Authors:** Luc Dedieu, Carole Serveau-Avesque, Stéphane Canaan

**Affiliations:** CNRS - Aix-Marseille Université - Enzymologie Interfaciale et Physiologie de la Lipolyse - UMR 7282, Marseille, France; University of Delhi, India

## Abstract

The enzymes belonging to the cutinase family are serine enzymes active on a large panel of substrates such as cutin, triacylglycerols, and phospholipids. In the *M. tuberculosis* H37Rv genome, seven genes coding for cutinase-like proteins have been identified with strong immunogenic properties suggesting a potential role as vaccine candidates. Two of these enzymes which are secreted and highly homologous, possess distinct substrates specificities. Cfp21 is a lipase and Cut4 is a phospholipase A_2_, which has cytotoxic effects on macrophages. Structural overlay of their three-dimensional models allowed us to identify three areas involved in the substrate binding process and to shed light on this substrate specificity. By site-directed mutagenesis, residues present in these Cfp21 areas were replaced by residues occurring in Cut4 at the same location. Three mutants acquired phospholipase A_1_ and A_2_ activities and the lipase activities of two mutants were 3 and 15 fold greater than the Cfp21 wild type enzyme. In addition, contrary to mutants with enhanced lipase activity, mutants that acquired phospholipase B activities induced macrophage lysis as efficiently as Cut4 which emphasizes the relationship between apparent phospholipase A_2_ activity and cytotoxicity. Modification of areas involved in substrate specificity, generate recombinant enzymes with higher activity, which may be more immunogenic than the wild type enzymes and could therefore constitute promising candidates for antituberculous vaccine production.

## Introduction

Tuberculosis (TB), which is one of the main causes of death from infectious diseases, accounted for 1.4 million deaths in 2011 (World Heath Organisation, Global Tuberculosis report 2012, http://www.who.int/tb/publications/global_report/en/index.html). More than 2 billion people are infected by *Mycobacterium tuberculosis,* the etiologic agent responsible for the disease, and 8 million people develop active TB every year. Efforts to fight *M. tuberculosis* are in progress, but no really efficient treatment has yet been found. In addition, the multi-drug and extensively drug-resistant strains which have emerged often result in therapeutic failure [1]. After the infection process, *M. tuberculosis* can survive in the host inside structures called granulomas that enable the bacteria to escape the host’s immune system [2]. During the process of granuloma formation induced by bacterial lipids, the macrophages become foamy and the lipids accumulating in their cytoplasm can be used by the bacteria as a carbon source [3], [4], [5], [6]. The mechanism whereby lipids are transferred from the host to the pathogen has not yet been completely elucidated, but recent results have suggested that lipolytic enzymes may play a major role in the life cycle of the mycobacteria [7], [8], [9]. Further characterization of the *M. tuberculosis* lipolytic enzymes should therefore lead to the development of new strategies for treating tuberculosis. Many lipolytic enzymes have been identified in the genome sequence of *M. tuberculosis,* including lipases, phospholipases, and more surprisingly, proteins related to cutinases which normally occur in phytopathogenic organisms [10], [11], [12].

Cutinases are extracellular enzymes which are involved in the virulence of phytopathogenic bacteria and fungi because they hydrolyse cutin, a polyester protecting plant leaves from evaporation and various aggression [13]. These enzymes belong to the α/β hydrolase fold family and contain a conserved “Ser, Asp, His” catalytic triad. In the cutinase protein family, the cutinase from the phytopathogenic fungi *Fusarium solani pisi* has been the most thoroughly studied in terms of its biochemical and structural characterization [14]. This enzyme is active on several substrates including cutin, triacylglycerols (TAG) and phospholipids [15], [16]. Although *M. tuberculosis* does not naturally encounter cutin polymers, seven genes (*Rv1758* or *Cut1*, *Rv1984c* or *Cfp21*, *Rv2301* or *Cut2*, *Rv3451* or *Cut3*, *Rv3452* or *Cut4*, *Rv3724* or *Cut5* and *Rv3802c* or *Cut6*) encoding proteins belonging to the cutinase family have been identified in the genome of *M. tuberculosis* H37Rv, and the proteins showed amino acid sequence identity ranging from 18 to 26% with the cutinase from the *F. solani pisi* amino acid sequence [17]. It has been established that these enzymes have several possible physiological functions, ranging from the cell wall formation to the cytotoxic effects on host cells. All the recombinant proteins have been previously produced and purified, and unlike the cutinase from *F. solani pisi*, none of these *M. tuberculosis* enzymes have been found to show cutinase activity [18]. Cut6 (Rv3802) shows phospholipase and thioesterase activity, in line with its physiological function as an essential enzyme involved in mycolic acid synthesis [19], [20], [21]. Despite the existence of 52% identity and more than 66% homology between Cfp21 (Rv1984) and Cut4 (Rv3452), these genes do not posses the same substrate specificities. Cfp21 is a lipase which hydrolyses mono and triglycerides, whereas Cut4 behaves like a phospholipase A with cytotoxic effects on macrophages [7]. Three specific areas which have been identified in the vicinity of the active site are probably involved in the substrate binding process, and could explain the substrate specificities of these enzymes [7]. In addition, studies on secreted cutinases such as Cfp21 and Cut4 have shown that these enzymes could be used as biomarkers in patients with active tuberculosis and therefore constitute interesting potential candidates for use in the production of vaccines [22], [23]. In view of these data, these enzymes are worth investigating more closely, since they could provide useful targets to diagnose and fight the disease.

In the present study, specific residues of Cfp21 identified by 3-dimensional structural models overlay were replaced by site-directed mutagenesis with residues occurring in Cut4 at the same locations. Seven mutant proteins, including 3 single mutants, 3 double mutants and a triple mutant, were overexpressed in *E. coli*, purified and characterized biochemically. Interestingly, two Cfp21 mutants showed higher rates of lipase activity than the wild type, and three mutants acquired phospholipase A activity and their cytotoxic effects on macrophages were found to be as efficient as that of Cut4. This study has made it possible to identify the amino-acids involved in the substrate binding of both cutinase enzymes and sheds interesting light on their distinct substrate specificities. The mutant proteins obtained here showed higher Specific Activities (SAs) on TAG and phospholipids than the wild type enzymes and constitute interesting potential candidates for vaccine production as well as useful tools for further studies on the physiological role of these cutinases involved in the lipid metabolism in *M. tuberculosis*.

## Materials and Methods

### Materials

*E. coli* DH10B strain was from Life technologies (Saint Aubin, France) and *E. coli* Rosetta(DE3)pLysS cells were purchased from Novagen (Darmstadt, Germany ). LB powder, Ni^2+^-nitrilotriacetic acid (NTA) agarose gel, fluorescent pyrene-derivative triglycerides and fluorescent phospholipids (PED-A_1_ and PC-A_2_) were from Life technologies. IPTG (Isopropyl β-D-1-thiogalactopyranoside), Tris (tris-hydroxymethyl-aminomethane), and ampicillin were obtained from Euromedex (Souffelweyersheim, France). CHES (N-Cyclohexyl-2-aminoethanesulfonic acid) was from Alfa Aesar (Karlsruhe, Germany). Vinyl-esters were purchased from TCI Europe (Zwijndrecht, Belgium), PLA_1_ from *Thermomyces lanuginosus* and pancreatic porcine PLA_2_ (ppPLA_2_) were purchased from Sigma-Aldrich (Saint-Quentin Fallavier, France). RPMI1640 culture medium and Fetal Calf Serum (FCS) were from PAA (Les Mureaux, France). The « Cytotox 96 nonradioactive cytotoxicity assay kit » used in the cytotoxicity assays was purchased from Promega (Charbonnieres, France).

### Methods

#### Construction of recombinant plasmids, expression and purification of recombinant proteins

Mutagenesis was performed by PCR using the QuikChange® system (Stratagene, La Jolla, CA, USA). PCR mutagenesis was performed with the primers described in Table 1 on the plasmid pDest14-*Cfp21* previously described [7]. This plasmid contains a nucleotide sequence coding for an N-terminal His_6_-tag followed by the TEV protease recognition site, followed by the mature Cfp21 protein. Seven plasmids (pDest-*Cfp21-A1*; -*A2*; -*A3*; -*A1A2*; -*A1A3*; -*A2A3*; and the triple mutant -*TM*) (Table 1) were generated and transformed in the *E. coli* DH10B strain (Life technologies, Invitrogen). The DNA sequences of all the plasmids were analyzed using DNA sequencing methods (GATC, Germany).

**Table 1:** Primers and plasmids used in this study.

Primers	Sequence 5′ → 3′	Aim
Cfp21-D54R-Fwd	cttctggtcttggc**cgc**gtcggtgaggcgttc	A1 mutants
Cfp21-D54R-Rev	gaacgcctcaccgac**gcg**gccaagaccagaag	A1 mutants
Cfp21-GDFL-Fwd	gaactacccagcaagcg**gc**gact**t**cc**t**cgcgagcg	A2 mutants
Cfp21-GDFL-Rev	cgctcgcg**a**gg**a**agtc**gc**cgcttgctgggtagttc	A2 mutants
Cfp21-RWR-Fwd	gcaccggaggcggc**cg**t**tgg**a**g**ggcgcatgtttcgtatgttca	A3 mutants
Cfp21-RWR-Rev	tgaacatacgaaacatgcgcc**c**t**cca**a**cg**gccgcctccggtgc	A3 mutants
**Plasmids**	**Description**	
pDest14-*Cfp21-A1*	pDest14-*Cfp21* mutated to encode Arg instead of Asp in position 54 of Cfp21 (A1 mutant)	
pDest14-*Cfp21-A2*	pDest14-*Cfp21* mutated to encode Gly-Asp-Phe-Leu instead of Asp-Asp-Tyr-Arg in position 90 to 93 of Cfp21 (A2 mutant)	
pDest14-*Cfp21-A3*	pDest14-*Cfp21* mutated to encode Arg-Trp-Arg instead of Asn-Ile-Met in position 183 to 185 of Cfp21 (A3 mutant)	
pDest14-*Cfp21-A1A2*	pDest14-*Cfp21* with A1 and A2 mutations	
pDest14-*Cfp21-A1A3*	pDest14-*Cfp21* with A1 and A3 mutations	
pDest14-*Cfp21-A2A3*	pDest14-*Cfp21* with A2 and A3 mutations	
pDest14-*Cfp21-TM*	pDest14-*Cfp21* with the 3 mutations (A1, A2, and A3) named TM (triple mutant)	

Nucleotides of the primers that differ from the wild type sequence are presented in bold.

Cut4 and Cfp21 proteins were produced, purified and refolded as described previously [7], and yielded 20 to 25 mg of protein per liter of culture (Table 2). All the mutant plasmids were transformed in the *E. coli* Rosetta(DE3) pLysS strain to produce recombinant enzymes in the form of inclusion bodies. In the case of the Cfp21 mutant proteins, the first step in the purification on a Ni^2+^-NTA column was performed in 8 M urea as previously described in the case of the wild type strain [7]. Each mutant was refolded in specific refolding buffers (Table 2). After the refolding process, soluble protein samples were concentrated on an ultrafiltration membrane (MWCO = 10 kDa, Amicon) and loaded onto a size-exclusion chromatography column (Superdex 200) equilibrated with the same buffer as that used in the refolding process (Table 2). The fractions corresponding to the elution volume of a monodisperse protein were pooled and concentrated. The His_6_-tag occurring in the N-terminal position was then removed by performing TEV proteolytic cleavage (overnight at 4°C, TEV/rCfp21 protein ratio 1/10) and protein without His_6_-tag was purified by excluding it from a Ni^2+^-NTA column. The molecular mass, the extinction coefficient at 280 nm and the isoelectric point including the His_6_-tag of Cfp21, Cfp21 mutants, and Cut4 were obtained from the ProtParam tool (http://ca.expasy.org/tools/protparam.html). Proteins were concentrated to 1 mg/ml and stored at –80°C.

**Table 2:** Refolding buffers and amount of purified protein per liter of culture medium obtained with the 7 Cfp21 mutant proteins in comparison with the Cfp21 and Cut4 wild type proteins.

	Refolding buffer	Amount of protein per culture liter (mg)
Cfp21-WT	Tris 10 mM, NaCl 150 mM, pH8	25
Cfp21-A1	CHES 50 mM, NaCl 150 mM, pH9	15
Cfp21-A2	Tris 10 mM, NaCl 150 mM, pH8	20
Cfp21-A3		20
Cfp21-A1A2	CHES 50 mM, NaCl 300 mM, pH9	20
Cfp21-A1A3		15
Cfp21-A2A3	CHES 50 mM, NaCl 150 mM, pH9	25
Cfp21-TM		5
Cut4	CHES 50 mM, pH9	20

#### Circular dichroism (CD)

Secondary structures of the Cfp21 mutant proteins were assessed using circular dichroism (CD) method as previously described in the case of the wild type Cfp21 protein [7]. Briefly, CD spectra were recorded on proteins in 250 µl of 10 mM phosphate buffer at pH 8, at 20°C on a Jasco J-81 dichrograph (Jasco, Milano, Italy) using 1-mm thick quartz cells. CD spectra were recorded between 195 and 245 nm at 0.2 nm/min. Mean values were obtained from three independent acquisitions. The buffer spectrum was subtracted from the experimental spectra and the resulting spectra were smoothed using the “mean-movement” procedure described in the Jasco Spectra Manager package. Mean residual molar ellipticity values (θ) were calculated as follows: θ = 3300×*m*×Δ*A*/(*l*×*c*×*n*), where *m* is the protein molecular mass, Δ*A* is the difference between right and left circularly polarized light, *l* is the path length (0.1 cm), *c* is the protein concentration (between 0.1 and 0.2 mg/mL) and *n* is the number of residues. The experimental data obtained between 195 and 245 nm were analyzed using the CDNN software program [24] to calculate the percentage of each type of secondary structure in the proteins.

#### Esterase and lipase assays using vinyl-esters and fluorescent triglycerides substrates

Enzymatic hydrolysis of solutions and emulsions of various (vinyl-) esters was monitored potentiometrically at 37°C for at least 5 min using a pH-Stat (Metrohm 718 STAT Titrino) [25]. Assays were performed at pH 8 in 20 ml of 2.5 mM Tris, 150 mM NaCl, and 0.25 mM NaTDC. The fatty acids released were automatically titrated by the pH-Stat with 0.1 M NaOH, and the enzymatic reaction slopes were recorded. Enzymatic activities were calculated from the slopes and expressed in µmole of fatty acid released per minute (U). Specific Activities (SA) are expressed in U per mg of protein (U.mg^−1^).

Lipase activity was assayed using a pyrene derivative triglyceride (1,2-dioleoyl-3-(1-pyren-1-yl)dodecanoyl-*rac*-glycerol) purchased from Life Technonolgies. Substrate stock solution (18.5 µM) was prepared in activity buffers. All enzyme activities were assayed at pH 8.0 in 10 mM Tris, 300 mM NaCl, and 4 mM NaTDC, except for Cut4, which was not stable in this buffer and was assayed at pH 9.0 in 50 mM CHES and 4 mM NaTDC. Enzymatic reactions were performed at 20°C for 15 min in a final volume of 200 µL containing 10 µg enzyme and 1.85 µM substrate. The rate of pyrene fluorescence released was recorded at λ_exc_ = 340 nm and λ_em_ = 380 nm using a 96-well plate fluorometer (Fluoroskan ascent, Thermoscientific). Enzymatic activities were quantified using a pyrene (Sigma-Aldrich, Saint-Quentin Fallavier, France) calibration curve (0.1–50 pmoles in activity buffers), and expressed in pmol of fatty acid (or pyrene) released per minute per mg of protein (pmol.min^−1^.mg^−1^).

#### Phospholipase A assays using radioactive and fluorescent substrates

Radioactive substrates and products: [1-^14^C] palmitic acid (200 µCi/ml), 1-palmitoyl-2-[1-^14^C] palmitoyl phosphatidylcholine ([^14^C]-DPPC, 25 µCi/ml) and 1-[1-^14^C] palmitoyl lyso-phosphatidylcholine (25 µCi/ml) were purchased from GE Healthcare. The amounts of enzyme used were 100 µg for wild type and mutants of Cfp21 and 10 µg for ppPLA_2_. In each assay, 50 nCi (110,000 dpm) of either [^14^C]-DPPC for PLA activity or [^14^C]-palmitoyl lyso-phosphatidylcholine for lysophospholipase activity were mixed with enzymes in a final volume of 250 µl in 50 mM Tris/HCl (pH 8), 100 mM NaCl, 5 mM CaCl_2_, 4 mM NaTDC. The total radioactivity was quantified using 20 µl of the total reaction mixture added to ULTIMA Gold scintillation liquid (Perkin-Elmer) and counted in a Beckman Scintillation counter LS1801. 230 µL of the preparation were then incubated at 25°C under vigorous shaking for 48 h and the reactions were stopped by adding 40 µl of 0.1 N HCl. After the 48-h incubation period, lipids were extracted twice with 150 µl of CHCl_3_/MeOH (2/1, v/v) and 25 µl were added to scintillation liquid and counted to determine the extracted radioactivity. The lipolysis products were then spotted onto silica plates (Merck, TLC Silica gel 60) along with standard amounts of [^14^C]-palmitic acid and [^14^C]-lysophospholipid and analyzed by performing thin layer chromatography (TLC), using two consecutive migrations with two different solvent mixtures, CHCl_3_/MeOH/H_2_O (65/25/4, v/v/v) on the first half of the plates and Hexane/Diethylether/Acetic Acid (80/20/1.5, v/v/v) up to the top of the plates. Plates were dried and exposed for 3 days in a dark room to a BioMax® MR-1 film (Kodak). Using the film, the radioactive spots corresponding to non hydrolysed DPPC, lysophospholipids, and free fatty acids were located on the plate, cut out and added to the scintillation liquid to be counted. To determine the PLA activity, three ratios were calculated in order to assess the percentage of DPPC hydrolyzed and that of the free fatty acids and lysophospholipids released: [radioactivity of DPPC or free fatty acids or lysophospholipids/(radioactivity of free fatty acids+radioactivity of DPPC+radioactivity of lysophospholipids)] ×100. This ratio gives the percentage of each molecular species released at the end of the reaction by the initial DPPC substrate.

The activities of recombinant proteins were also assayed using highly sensitive fluorogenic phospholipid substrates. Phospholipase A_1_ and A_2_ activities were monitored continuously using BODIPY® dye-labeled phospholipids: PED-A_1_ (N-((6-(2,4-DNP)Amino)Hexanoyl)-1-(BODIPY® FL C5)-2-Hexyl-Sn-Glycero-3-Phosphoethanolamine) and red/green BODIPY® PC-A_2_ (1-O-(6-BODIPY-®558/568-Aminohexyl)-2-BODIPY®FLC5-Sn-Glycero-3-Phosphocholine), respectively [26], [27]. The PED-A_1_ substrate has a BODIPY® FL fluorescent moiety conjugated at the *sn*-1 position and contains a dinitrophenyl group conjugated to the polar head group to provide intramolecular quenching. When the activity of a PLA_1_ hydrolyzes the *sn*-1 fatty acyl chain, the quenching process is therefore cancelled and the fluorescent signal is measured. The *sn*-2 fatty acyl group in PED-A_1_ is a non hydrolyzable alkyl chain, and PED-A_1_ substrate was used to specifically measure the activity of PLA_1_ enzymes. The red/green BODIPY® PC-A_2_ is a glycerophosphocholine with a BODIPY 558/568 dye–labeled *sn*-1 uncleavable alkyl chain and a BODIPY® FL dye–labeled *sn*-2 acyl chain. The cleavage at the BODIPY® FL dye–labeled *sn*-2 position resulted in a decrease in the rate of quenching by FRET (fluorescence resonance energy transfer) in the BODIPY® 558/568, resulting in an increase in the BODIPY® FL fluorescence. Substrate stock solutions (50 µM) were prepared in ethanol. All enzyme activities were assayed at pH 8.0 in 10 mM Tris 300 mM NaCl, except for Cut4, the activity of which was measured at pH 9.0 in 50 mM CHES. Enzymatic reactions were performed at 20°C for 15 min in a final volume of 200 µL containing 10 µg enzyme and 5 µM substrate (final concentration). The release of BODIPY® (BFCL5) (Life Technologies) was recorded at λ_exc_ = 485 nm and λ_em_ = 538 nm using a 96-well plate fluorometer (Fluoroskan ascent, Thermoscientific). Enzymatic activities were quantified using a BFCL5 calibration curve (0.08–200 pmoles in activity buffers) and expressed in pmol of fatty acid (or BFLC5) released per minute per mg of protein (pmol.min^−1^.mg^−1^). PLA_1_ from *Thermomyces lanuginosus* (Sigma-Aldrich, Saint-Quentin Fallavier, France) was used as a positive standard to determine the PLA_1_ activity, and ppPLA_2_ (Sigma-Aldrich, Saint-Quentin Fallavier, France) to determine the PLA_2_ activity.

#### Cytotoxic effects on mouse macrophage cells

The cytotoxic effects of the Cfp21 mutant proteins on mouse macrophages were determined using the mouse macrophage cell line RAW264.7 (ATCC number TIB-71). RAW264.7 cells were routinely grown in RPMI 1640 culture medium supplemented with 10% of Foetal Calf Serum (FCS) in the presence of 5% CO_2_, at 37°C in a humidified atmosphere. Prior to the cytotoxic assays, macrophages were inoculated in 24-well plates and grown to cell confluence (1×10^6^ cells/well) in RPMI 1640 culture medium supplemented with 5% of FCS. Macrophage cell confluence was assessed microscopically and enzymes were then added to each well (10 µg with the ppPLA_2_ and 100 µg with all the other proteins). These experiments were repeated with buffer alone (without any protein), THL inhibited protein, and heat denatured protein. After 16 and 24 hrs, cellular damage and morphological changes in the macrophage cells were observed microscopically (magnification 200 X) and the cytotoxicity of the enzymes was assessed by measuring the lactate dehydrogenase (LDH) activity released from the cytosol of damaged cells into the culture medium. Experiments were performed in line with the manufacturer’s instructions (Promega Kit CytoTox 96® Non-Radioactive Cytotoxicity Assay). Each condition was assayed in triplicate and repeated three times. Relative cytotoxicity was expressed as the rate (%) of cell lysis, and calculated from the LDH activity released from enzyme treated cells subtracted from that of buffer treated cells as a percentage of the total amount of LDH present in the fully lysed positive standard cells provided in the kit.

#### Inhibition studies with THL

Inhibition experiments with tetrahydrolipstatin (THL) were carried out using a lipase/inhibitor preincubation method [28]. Briefly, enzymes were pre-incubated for 60 min at 25°C with THL initially solubilized in Dimethyl-Sulfoxyde (DMSO) at an enzyme:inhibitor molar ratio of 1∶200. DMSO volume was always less than 10% of the total volume, and a negative control was performed with the same volume of DMSO without any inhibitor. Residual activity and cytotoxic effects were then assayed as described above.

### *In silico* Protein Modeling and Ligand Docking

Three-dimensional structural models of wild type Cut4 and wild type or mutant Cfp21 proteins were generated with the automatic protein structure homology modeling server using the I-Tasser software program [29], [30]. Although the models start after the signal peptide [7], the residue numbering of the full length protein has been used in the text. The structural overlay and figure were drawn using the PyMOL Molecular Graphics System (version 1.3, Schrödinger, LLC). Molecular docking was performed with the Autodock Vina program, and the results were interpreted using its plugin for PyMOL under Windows XP [31]. TAG and phospholipid ligand molecules were generated and their geometries were refined using the open source Avogadro software program (Version 1.0.1. http://avogadro.openmolecules.net/). Docking runs were performed after replacing the catalytic serine by a glycine in the receptor proteins so that the ligand would have access to its putative position during the reaction in the transition state model. The box size used was chosen to fit the whole cleft in the active site and rule out non constructive binding positions elsewhere on the protein surface.

## Results

### Three Dimensional Models, Site-directed Mutagenesis and Protein Purification

**Figure 1:**
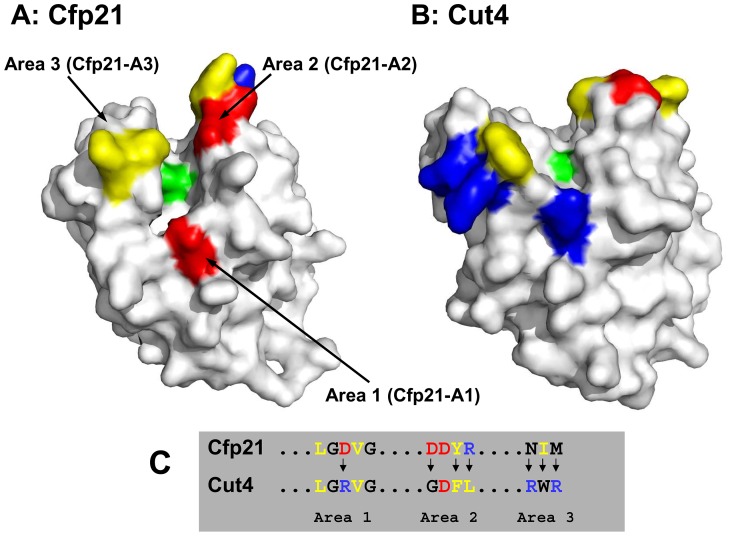
Mapping of the main differences between the Cfp21 and Cut4 structural models in the vicinity of the active site. (A) Cfp21 model. (B) Cut4 model. (C) Amino acid sequences in the 3 mutated areas. Only the amino acids in the three mutagenized areas (A1, A2, and A3) are coloured. Hydrophobic amino acids are in yellow. Negatively charged amino acids are in red and positively charged amino acids are in blue. Residues Asp^54^ in area 1 (A1), Asp^83^, Tyr^85^, Arg^86^ in area 2 (A2), and Asn^189^, ^Ile^190, Met^191^ in area 3 (A3) present on Cfp21 were replaced using site-directed mutagenesis methods by residues found to exist in Cut4 at the same location in the structural models. The amino acids are numbered starting with the initial methionine in the same way as for a protein precursor including the signal sequence.

Despite the high level of homology known to exist between their amino acid sequences, the biochemical data obtained on Cfp21 and Cut4 indicated that these two proteins have distinct substrate specificities [7] showing that Cfp21 is a lipase and Cut4 is a strict phospholipase. In order to understand this difference, Cfp21 and Cut4 structural models (Figure 1A–B) were built, based on the crystal structure of the *Fusarium solani* cutinase (pdb code 1CEX) [32], using the I-Tasser Server software program [30]. The superimposition of the cutinase 3D structure and Cfp21 and Cut4 models showed good alignment of the α/β hydrolase fold structural elements (α-helices located on either side of the central β-sheet) and the catalytic triads composed of Ser/Asp/His residues in each protein. Interestingly, this structural overlay in the models revealed that three sequence regions located in the vicinity of the active site, which we have called A1, A2 and A3, differ markedly between the three proteins. *In silico* molecular docking studies showed that these three regions are close to the active site and able to interact with the transition states of a TAG glyceroltricaprate (data not shown), and thus confirmed the choice of these areas in the subsequent site-directed mutagenesis analyses.

**Figure 2:**
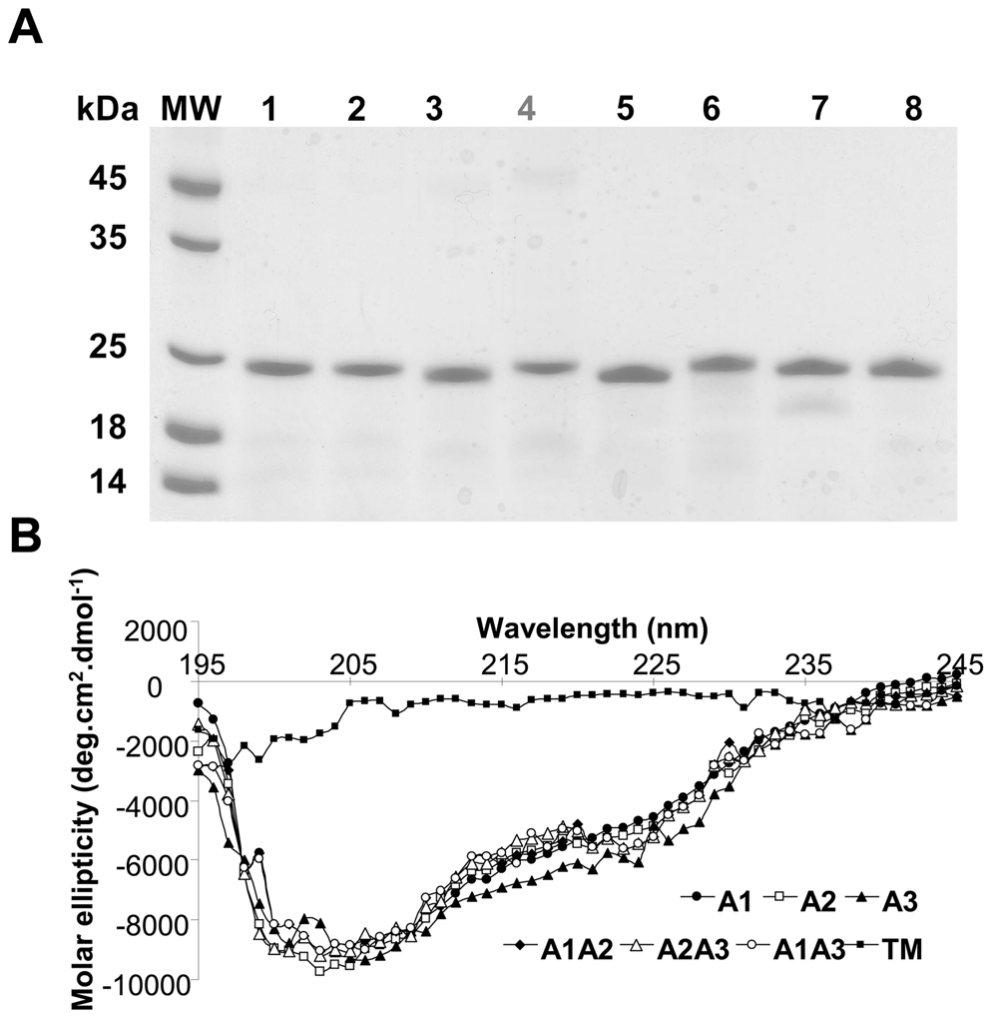
SDS-PAGE and CD spectra analysis of purified proteins. A) Protein purity assessed on SDS-PAGE. MW: molecular weight standard from Euromedex; 1: Cfp21 WT; 2: Cfp21-A1; 3: Cfp21-A2; 4: Cfp21-A3; 5: Cfp21-A1A2; 6: Cfp21-A2A3; 7: Cfp21-A1A3; 8: Cfp21-TM. 1 µg of each protein was loaded onto a 15% polyacrylamide gel and stained with Coomassie Blue. B) Far UV spectra of the Cfp21 mutant proteins.

The main goal of this study was therefore to elucidate how these sequence differences contribute to the phospholipase activity observed in Cut4. Due to the difficulties encountered in refolding Cut4 in our previous study [7], we decided to systematically replace the Cfp21 amino acids by amino acids having the same locations in the Cut4 model in order to obtain Cfp21 mutated proteins. The Asp^54^ residues present in region A1, the Asp^83^, Tyr^85^, and Arg^86^ residues present in region A2, and the Asn^189^, Ile^190^, and Met^191^ residues present in region A3 of Cfp21 were replaced by residues Arg^66^ (A1), Gly^94^, Phe^96^, and Leu^97^ (A2), Arg^203^, Trp^204^, and Arg^205^ (A3) of Cut4, respectively (Figure 1C). The mutations introduced allowed to reverse the charge from negative to positive in region A1, to induce the loss of a negative and a positive charge in region A2, and to add two positive charges in region A3. Seven plasmids were generated using the site directed mutagenesis method (Table 2). Three of them encoded proteins with one mutated region (Cfp21-A1, -A2, and -A3), 3 encoded proteins with 2 mutated regions (Cfp21-A1A2, -A1A3, and -A2A3) and one encoded a triple mutant (Cfp21-TM). As with the wild type Cfp21 enzyme (Cfp21-WT), all seven variants of the enzyme were produced and purified in the form of inclusion bodies at about 100 mg per liter of culture medium in *E. coli* Rosetta(DE3)pLysS (Table 2). Purified inclusion bodies were solubilised in 8 M urea and refolded using the same dilution method as with Cfp21-WT (see the Materials and Methods section). Although single mutants Cfp21-A2 and Cfp21-A3 were optimally refolded using the same buffer as for the Cfp21-WT protein, Cfp21-A1 mutant, all the double mutants and the triple mutant were more efficiently refolded in 50 mM CHES (N-Cyclohexyl-2-aminoethanesulfonic acid) buffer containing 150 or 300 mM NaCl at pH 9 (Table 2). The latter buffers are similar to the unique refolding buffer which is used to obtain an active Cut4 (50 mM CHES at pH 9) [7] but containing sodium chloride. After the refolding process, all the proteins were further purified by performing size-exclusion chromatography. The fraction corresponding to the elution volume of a monodisperse protein were pooled, concentrated, and analyzed by performing SDS-PAGE (Figure 2A). Between 15 and 25 mg of refolded mutant proteins were obtained per liter of culture medium, as occurred previously with Cfp21-WT [7], with the exception of refolded Cfp21-TM, where the amount obtained was 5 times lower. In the latter case, the refolding was not optimal, and only the buffer specified in Table 2 yielded the protein in a soluble form; under all the other 95 conditions tested, the protein precipitated [33]. Subsequent size-exclusion chromatography revealed that more than 70% of the protein was aggregated (data not shown).

The correct refolding of each recombinant protein was checked by performing circular dichroism (Figure 2B). These structural data showed that except for Cfp21-TM, all the recombinant proteins were properly folded and that 29 to 31% of their components were α-helices, 15 to 18% were β-sheets and 40 to 42% were random coils, as previously observed in the case of the Cfp21-WT protein [7]. In the case of Cfp21-TM alone, the circular dichroism data showed that the protein was unfolded, even in the soluble, non aggregated form obtained with the most efficient buffer.

### Changes in the Esterase and Lipase Activities

The seven mutants were characterized biochemically, using vinyl esters with alkyl chains ranging from 4 to 16 carbon atoms and fluorescent pyrene-labelled triglycerides with chains 18 carbon atoms long. Using vinyl esters as substrates (Table 3), single mutants Cpf21-A1, -A2, -A3 and double mutants Cfp21-A1A2, -A1A3 and -A2A3 showed a strong preference for substrates with a medium chain length ranging between 6 and 8 carbon atoms, such as Cfp21-WT. However, the specific activities (SA) values recorded depended on the mutated area: the A2 mutation was responsible for an increase in activity of 17 to 18% in comparison with Cfp21-WT when VC6 and VC8 were used as substrates, the A1 mutation decreased the activity of the protein by 31% and 25% on the same substrates, and the Cfp21-A3 mutant lost 99% and 80% of its activity. In addition, all the double mutants were significantly less active than the single one, even in the case of the combination containing the A2 mutation.

**Table 3:** Esterase specific activities (SA) of the Cfp21 mutant proteins.

	VC4	VC6	VC8	VC10
Cfp21-WT	68±6	2069±24	2103±62	45.8±10
Cfp21-A1	28±2	650±68	520±45	12±1.5
Cfp21-A2	87±7	2433±32	2500±55	75±6
Cfp21-A3	7±1	25±3	410±30	12±0.8
Cfp21-A1A2	16±1.4	64±5	125±11	17±0.8
Cfp21-A1A3	7±0.3	19±3	12±1	2±0.4
Cfp21-A2A3	6±1	26±2	62±5	8±1.2
Cfp21-TM	ND	ND	ND	ND

The SAs of all the recombinant enzymes obtained using Vinyl-esters were determined by titrating the free fatty acids released using the pH-Stat technique and expressed in international units per mg of protein (U/mg). Vinyl esters with alkyl chains ranging from 4 to 16 carbon atoms were assayed. Only substrates with alkyl chains ranging from 4 to 10 carbon atoms are presented, since no significant activity was detected with longer alkyl chains.

All substrates were assayed at concentrations above their solubility limits as previously described [46]. ND: not detected.

**Figure 3:**
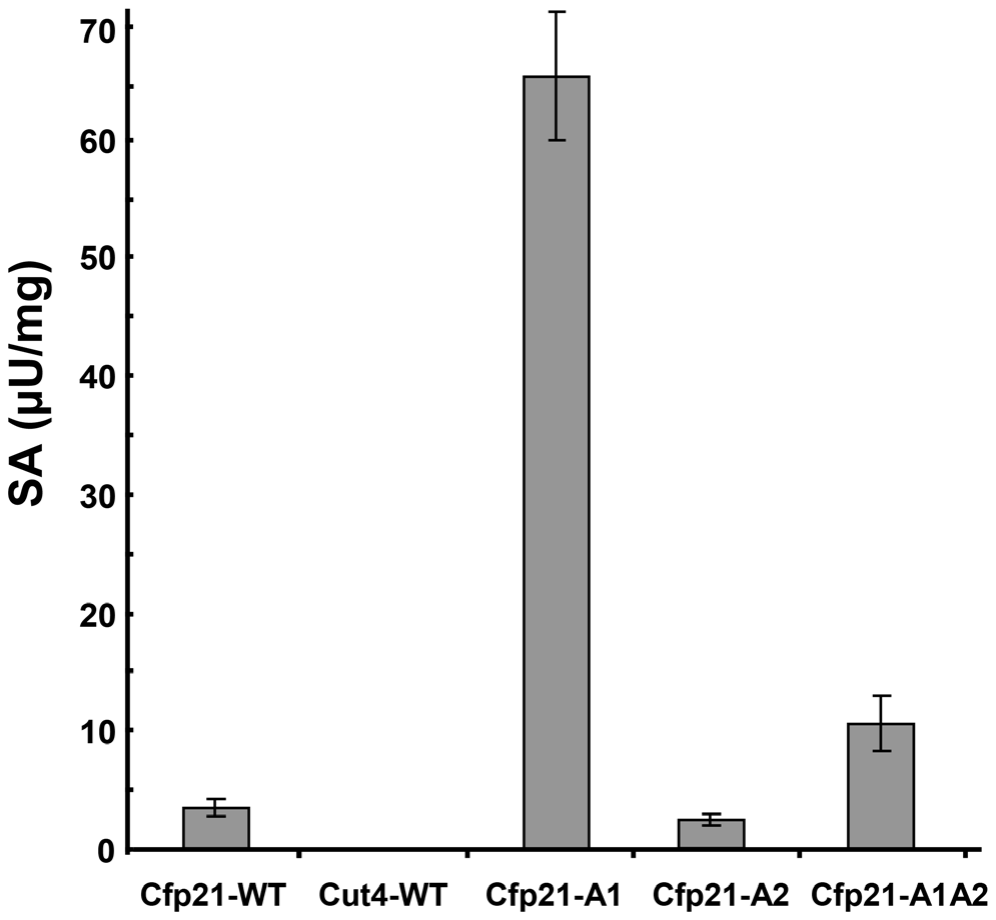
Lipase Specific Activities (SAs) of the Cfp21 mutants on fluorescent triglycerides. SAs were calculated from the velocity slope obtained for 10 minutes using 10 µg of enzymes. Results are the means obtained in at least 3 independent experiments. Only the mutants with lipase activity are presented. Cfp21 and Cut4 were used as positive and negative standards, respectively [7].

Using fluorescent pyrene-labelled triglyceride containing fatty acid with a long carbon chain, in comparison with the wild type strain (SA of 4 µU/mg), the A1 and A1A2 mutants were 15 fold and 2.5 fold more active, giving SA values of 65.5 and 10.5 µU/mg, respectively. Interestingly, the Cfp21-A1 mutant (65.5 µU/mg) was 15, 32 and 6.2 fold more active than the Cfp21-WT (4.3 µU/mg), Cfp21-A2 (2 µU/mg) and Cfp21-A1A2 (10.5 µU/mg), respectively (Figure 3). It is worth noting that the A1 mutation enhanced the lipase activity on triglycerides with long carbon chains and the A2 mutation with mono-ester substrates with short and medium carbon chains. All the mutants with the A3 mutation completely lost their lipase activity, in line with the results obtained with vinyl-esters. Cfp21-TM did not show any significant activity whatever the substrate used, which is consistent with the fact that this protein was in the unfolded state, as shown by the CD spectrum (Figure 2B).

### The Acquisition of Phospholipase A Activities by Cfp21 Mutant Proteins

**Figure 4:**
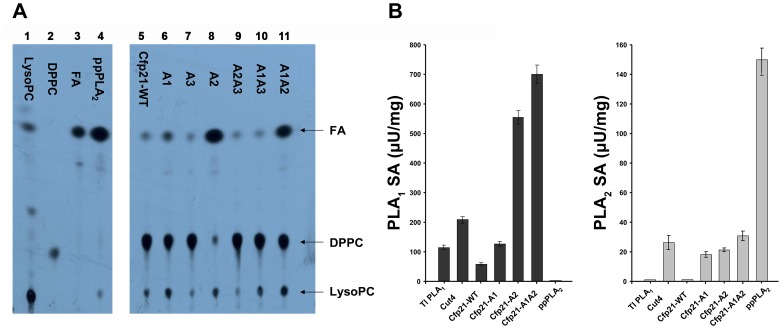
Phospholipase A activities of the Cfp21 mutants. A) phospholipase A activity of the Cfp21 mutants: Hydrolysis of ^14^C-DPPC. FA: fatty acid; DPPC: dipalmitoyl-phosphatidylcholine; LysoPC: palmitoyl-lyso-phosphatidylcholine. Pure LysoPC, DPPC and Palmitic acid (FA) were used as standards (lanes 1, 2, and 3). Pancreatic porcine phospholipase A_2_ (ppPLA_2_) was used as a positive standard (lane 4). Products resulting from a 24-h enzymatic reaction with wild-type Cfp21 and its mutants were loaded into lanes 5 to 11. Rates of hydrolysis were calculated as described in the experimental section. B) Specific activities of phospholipase A_1_ and A_2_ on fluorescent phospholipids. The PLA_1_ from *Thermomyces lanuginosus* was used as a positive standard to determine the PLA_1_ activities, and ppPLA_2_ was used as a positive standard to determine the PLA_2_ activities. The specific fluorescent substrates used to measure the PLA_1_ and PLA_2_ activities were BODIPY® dye-labeled PED-A1 and the red/green BODIPY® PC-A2, respectively, as described in the Material and Methods section. Specific activities were calculated from the velocity slope obtained for 10 minutes using 10 µg of enzymes. Results are means obtained in at least 3 independent experiments.

In order to assess the phospholipase activity of mutant proteins, hydrolysis of dipalmitoyl-phosphatidylcholine radiolabeled at the *sn*-2 position ([^14^C]-DPPC) was assayed. After 24 hours of incubation, although the positive control (ppPLA2) hydrolyzed 100% of the substrate, Cfp21-WT was not able to hydrolyze the DPPC since the rates of free fatty acids released did not exceed 4%, corresponding to the background value (Figure 4A) [7]. Likewise, none of the hydrolysis experiments performed with mutants containing the A3 mutation (Cfp21-A3, –A1A3 and -A2A3) resulted in any significant hydrolysis of the substrate; whereas low, medium, and strong rates of release of free fatty acids from the [^14^C]-DPPC were detected with Cfp21-A1, -A1A2, and -A2 (Figure 4A), reaching 9%, 41%, and 81%, respectively. These results suggested that these 3 mutants (Cfp21-A1, Cfp21-A2, and Cfp21-A1A2) clearly acquired PLA activities. In addition, Cfp21-A1, Cfp21-A1A2 and Cfp21-A2 released 14%, 16% and 13% of the radiolabeled lysoPC, respectively, during the hydrolysis of DPPC (Figure 4A). Since only a cleavage at the *sn*-1 position could account for the release of a radiolabeled lysophospholipid, these results indicate that these three mutants were able to hydrolyse not only at the *sn*-2 but also at the *sn*-1 position. However, these radioactive assays did not make it possible to quantify these activities in terms of the specific activity (SA). The phospholipase activity of Cfp21 mutants showing greater levels of activity was therefore further characterized using specific fluorescent phospholipids having non hydrolysable positions in either the *sn*-1 or the *sn*-2 position (see Material and Methods section). The use of these substrates made it possible to discriminate between the two types of phospholipase activities for all the proteins tested and to compare their specific activities. *Thermomyces lanuginosus* (*Tl*) PLA_1_ and ppPLA_2_ were used as positive PLA_1_ and PLA_2_ controls, respectively. In contrast to Cfp21-WT with which only a very slight PLA_1_ activity was detected (50 µU/mg), strong PLA_1_ activities were observed with Cfp21-A2 and Cfp21-A1A2, where the SA values reached 550 and 700 µU/mg, corresponding to an 11-fold and a 14-fold increase in activity in comparison with Cfp21-WT, respectively (Figure 4B). The PLA_1_ activity detected with Cfp21-A1 was 112 µU/mg, which is a twice higher level of activity compared to the low level recorded with Cfp21-WT (Figure 4B). Surprisingly, Cut4 also showed a PLA_1_ activity as high as 210 µU/mg, which is 4 times higher than that observed with Cfp21-WT. As far as the *sn*-2 position cleavage was concerned, Cut4 showed an apparent PLA_2_ activity (27 µU/mg) while Cfp21-WT did not hydrolyze this position on this substrate. Mutations performed in A1 and A2 areas resulted in SA values of 19 and 21 µU/mg for Cfp21-A1 and -A2, respectively, which are similar to SA values recorded with the Cut4 enzyme. In addition, the SA of the double mutant Cfp21-A1A2 was greater than those of the single mutants described above. As observed in the radioactive assay, mutants with the A3 mutation did not acquire any phospholipase activity whatever the substrate used. Taken together, these data clearly indicate that only the Cfp21-A1, A2, and A1A2 mutants acquired phospholipase A activities and particularly a strong phospholipase A_1_ activity.

### Cytotoxic Effects of Cfp21 Mutant Proteins on Mouse Macrophages

**Figure 5:**
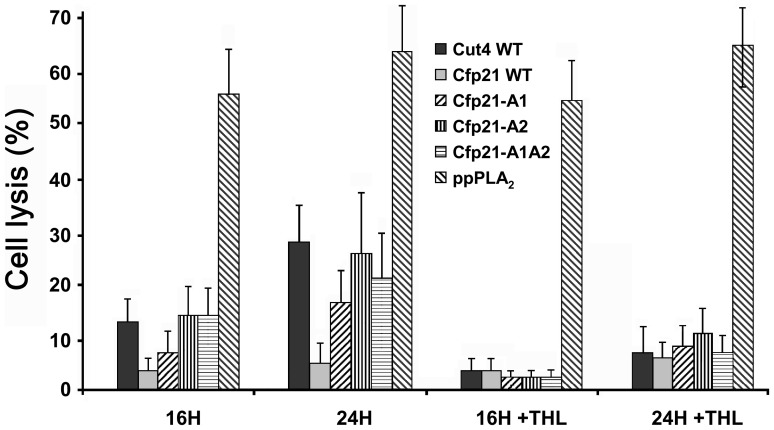
Cytotoxic effects of Cfp21 mutants on mouse macrophage cells. Cytotoxic effects causing macrophage lysis were monitored by measuring the release of LDH into the culture media. LDH activities were measured after 16 h and 24 h of incubation with 150 µg of *M. tuberculosis* enzymes or 10 µg of ppPLA_2_. The rate (%) of macrophage cell lysis was calculated as described in the experimental section. Similar experiments were performed using THL inhibited enzymes to determine the involvement of active serines in the cytotoxic process.

Contrary to Cfp21, Cut4 was found to have cytotoxic effects on macrophages, which suggests the role possibly played by this enzyme in host-pathogen relationships [7]. These cytototoxic effects were therefore investigated with the mutants Cfp21-A1, -A2, and -A1A2, which had acquired a strong lipase and/or phospholipase A activity. Under the same experimental conditions, these three mutants were found to have cytotoxic effects on macrophages, giving rates of cell lysis of up to 18%, 26% and 22% after 24 hours, respectively (Figure 5). These effects were comparable to those observed with Cut4, and remained lower than ppPLA2 (positive control), with which nearly 64% of cell lysis was reached after 24 hours (Figure 5) [34]. The morphology of the macrophages was also examined under light microscopy. Macrophages treated with Cut4 and mutants showed significant morphological changes and a loss of confluence (data not shown) correlated with the cell lysis measured by the LDH released. To confirm that these lytic effects were due to the activity of the enzymes added, similar experiments were carried out with either heat inactivated enzymes or enzymes inhibited using THL, which is known to be a specific serine enzyme inhibitor [7], [35]. Results showed that with all these mycobacterial enzymes, the release of LDH can be abolished either by preincubating the enzymes with THL (Figure 5) or by inactivating the recombinant enzymes by heat treatment (data not shown); whereas preincubating ppPLA_2_ with THL did not abolish the release of LDH (Figure 5), since THL does not inhibit the activity of secreted PLA_2_ [35].

## Discussion

Despite their initial annotation, cutinase-like proteins of *M. tuberculosis* H37Rv have very different catalytic properties from those of the well characterized *Fusarium solani pisi* (*Fs*) cutinase. None of them hydrolyse cutin and none of them have such a large spectrum of activity as the *Fs* cutinase [7], [16], [18]. It was previously established by our group that despite the 50% identity shown by their amino acid sequences, Cfp21 is a lipase and Cut4, a phospholipase [7]. In the present study, the substrate specificities of these enzymes were elucidated by identifying three specific areas in the vicinity of the active site of Cfp21. In addition, it was established how changes in a few amino-acids enhance the lipase activity, induce phospholipase activity and generate enzymes with cytotoxic effects, as efficient as Cut4.

It is worth noting that the buffers giving the most efficient refolding of the Cfp21-A1 mutant, all the double mutants and the triple mutant were composed of CHES at pH 9, which was also the case with Cut4 (Table 2). However, none of these buffers were exactly the same as that used with the Cut4 protein, since NaCl concentrations ranging between 150 and 300 mM were required for efficient refolding to occur. This finding indicates that the structure of the Cfp21 mutants located near the active site became increasingly similar to that of the Cut4 protein when double mutations were performed, and suggests that these slight modifications are crucial to the folding of the active site and the substrate specificity. Unlike Cut4, the Cfp21-WT and mutants were not denaturated by presence of NaCl in the buffer, which suggests that despite the mutations, the structure body of Cfp21 is more appropriate to tolerate high ionic strength in the buffers. In the case of the triple mutant, the CD spectra indicate unambiguously that the protein was completely unfolded, and that the inefficient refolding observed in Cut4 therefore originated mainly from these 3 areas (Figure 2B).

In terms of the substrate specificity, the *in silico* docking data obtained on TAGs and phospholipids in the two 3D wild type enzyme models indicated that the three areas identified by the structural overlay can interact with their specific substrates (data not shown). Mutations can therefore be expected to induce either a shift from lipase to phospholipase activity or the acquisition of phospholipase activity in addition to the lipase activity. Our biochemical data showed that all the three areas are involved in the substrate specificity since the three mutations clearly altered the substrate binding site of Cfp21. Indeed, the A3 mutant is active on mono-ester substrates but could not accommodate larger substrates and the A1, A2, and A1A2 mutants acquired phospholipase activity without any loss of lipase activity (Figure 3 and 4). This may be attributable to the fact that these mutants showed more widely open structures than Cfp21-WT in the *in silico* models (data not shown), thus presenting a wider cleft for welcoming the substrate. This cleft was more similar to that observed in the Cut4 3D model, in agreement with the increase in phospholipase B activity observed in these mutants. This is also in agreement with our experimental data showing that greater lipase activity occurred in the mutant when the *sn*-3 fluorescent pyrene-labelled triglyceride was used as a substrate. Although triglycerides are uncharged molecules, the change of charge observed in the A1 mutant favoured the lipase activity this particular mutant showed greater lipase activity than the Cfp21-WT, whereas the A2 mutation did not affect the level of activity of the mutant. By contrast, the A2 mutation was found to have strong negative effects on the lipase activity in the A1A2 mutant, probably because the substrate was poorly bound to the active site despite the presence of larger cleft.

Although all the mutants carrying the A3 mutation completely lost their lipase activity, the data obtained on vinyl esters with short, medium and long chains (Table 3) showed that the mutants were active specifically on these substrates, which chemically resemble to monoglycerides more closely than triglycerides. These results therefore indicate that all the mutants carrying this modification were still able to accommodate mono-ester substrates but lost the ability to accommodate substrates consisting of larger di or tri-esters, possibly because the acyl chain could not be stabilised in these mutants due to the lack of interactions.

The Asn^189^, Ile^190^, Met^191^ residues in Cfp21 are therefore essential, or at least more appropriate, for the lipase activity than the Arg^203^, Trp^204^, and Arg^205^ residues in Cut4, which is not surprising since i) the Tryptophane residue seems located inside the catalytic pocket preventing the interaction with long acyl chain substrates due to a possible steric hindrance and ii) the two arginine residues that are positively charged and belonging to the binding site of the protein but in the opposite side of the catalytic pocket could alter the interaction with the lipid-water interface. Moreover it cannot be excluded that the observed effects with the A3 mutants could be due to the vicinity of the Area 3 and the active site His^193^ residue, and thus catalytic properties could be modified for these mutants.

When a DPPC radiolabeled in the sn-2 position was used as a substrate for the Cfp21 mutants, radiolabeled free fatty acids and lysophospholipids were released and the PLB activity was detected, *i.e.,* both PLA_1_ and apparent PLA_2_ activities occurred. When highly sensitive and specific fluorescent substrates with non hydrolysable *sn*-*1* or *sn-2* alkyl chains [26], [27] were used, both activities were detected in the three Cfp21 mutants as well as in Cut4. As shown in figure 4B, a strict PLA_1_ activity was observed with the *Thermomyces Lanuginosa* PLA_1_ and a strict PLA_2_ activity with the ppPLA_2_. In addition, Cut4 harbours PLB activity (*i.e.,* PLA_1_ and PLA_2_ activities): the PLA_1_ activity was ten-fold greater than the apparent PLA_2_ activity. Cfp21-A1, -A2 and -A1A2 mutants also showed both activities, which were 6 fold, 27 fold, and 23 fold more active, respectively, in the case of the PLA_1_ *versus* apparent PLA_2_ activity. For PLB activity, it is clear that mutations performed in both areas A1 and A2 were responsible to phospholipid hydrolysis at the sn-2 position. Moreover, the SA of the double mutant Cfp21-A1A2 that is greater than those of the single mutants Cfp21-A1 and Cfp21-A2 alone, suggests that these mutations may have additive effects. The fact that rCut4 was also able to hydrolyse the PLA_1_ substrate was unexpected, since no radiolabeled lysophospholipid was observed in a previous study [7]. To explain this discrepancy, the lysophospholipase activity of Cut4 and Cfp21 mutants was assessed. Contrary to the Cfp21 mutants, a lysophospholipase activity was detected in Cut4 (data not shown). It was therefore concluded that during the 48-h incubation period prior to the radioactive assay, *sn*-1 and *sn*-2 cleavage occur with Cut4 and the three Cfp21 mutants, and radiolabeled lysophospholipids and fatty acids are released. In the case of Cut4, the lysoPLA_2_ activity may induce the release of radiolabeled fatty acids from the lysophospholipid, which would explain why no radiolabeled lysophospholipid was detected after 48 h of hydrolysis. In addition, during this long incubation experiment, non enzymatic reactions such as trans-esterification are known to possibly/sometimes occur, which may transfer the radiolabeled fatty acid from the sn-2 position to the sn-1 position of the substrate, where it might be hydrolyzed by the lysoPLA_1_ activity. This experimental artifact might result in *sn*-2 cleavage being over-estimated when this method is used, but the data obtained here using fluorescent substrates showed that the SA of PLA_1_ was greater than that of PLA_2_. Unlike PLA_2_ enzymes, PLA_1_ enzymes often show several activities [36], [37]. Moreover, many PLB were initially defined as PLA_1_ or PLA_2_ because the authors tested only one kind of substrate or because the methods used were not very sensitive. This was what happened in the case of first so-called PLA_1_ identified: the bacterial outer membrane phospholipase A (OMPLA) [38], which was initially described as a PLA_1_, was redefined 6 years later as a PLB/lysoPLA_1/2_ enzyme, since it can cleave at both the sn-1 and sn-2 positions of diacyl- or lysophospholipids [39]. Here, Cfp21 mutants and the wild type Cut4 enzymes showed PLB activity with a higher SA in the case of PLA_1_ *versus* PLA_2_. In addition, PLB and LysoPLA can tend to be confused because most PLBs possess lysophospholipase activity [40], as observed here in the case of cut4.

Lastly, the phospholipase activity is clearly responsible for the cytotoxicity of Cfp21 -A1, -A2, -A1A2 mutants as observed with Cut4. Many fully characterized cytolytic bacterial outer-membrane enzymes and secreted phospholipase C and phospholipase A_2_ are involved in virulence in many bacterial species such as *Pseudomonas aeruginosa, Listeria monocytogenes, Clostridium perfringens*, *Yersinia enterocolitica*, and *Campylobacter coli* [41], [42], [43], [44]. However, only a few PLA_1_ and PLB have been reported to possess a cytotoxic activity: the secreted PLA_1_ activity of the human pathogen *Yersinia enterocolitica* and the PL-A_1_, -A_2_, and -C activities of *Helicobacter pylori* are known, for example, to be virulence factors responsible for phospholipid degradation processes in mucosal barriers [44]. In mycobacteria, phospholipases C and Cut4, which were previously described as a PLA_2_, have been reported to have cytotoxic effects on macrophage cells [7], [45]. Here we describe the presence of a dual PLB activity in Cut4 and the Cfp21-A1, -A2, and -A1A2 mutants. In addition, cytotoxic effects on macrophages were observed in the presence of these four proteins and not the others. Experiments using negative standards with THL-inhibited and heat inactivated enzymes clearly showed that these cytotoxic effects were due to an enzymatic activity. However, since none of these proteins was either a strict PLA_1_ or a strict PLA_2_, it was not possible to determine which of these PLA activities was responsible for the cytotoxic effects observed on macrophage cells. However, the fact that the ppPLA_2_ was cytotoxic whereas the PLA_1_ from *Thermomyces lanuginosus* was not suggests that the apparent PLA_2_ activity may have been involved in the cytolytic effects observed. In addition, the PLA_2_ activities and the cytotoxic effects observed with Cut4 and Cfp21 mutants were in the same range, whereas the PLA_1_ activitiesdiffered considerably between the four proteins: the fact that the Cfp21-A2 and Cfp21-A1A2 mutants showed a much greater SA on PLA_1_, which was not accompanied by any increase in their cytotoxic effects in comparison with those of wild-type Cfp21 and the Cfp21-A1 mutant, is consistent with the above hypothesis.

The results obtained in this study account for the difference in substrate specificity observed between two cutinase enzymes showing more than 50% amino acid identity. A set of proteins with a broad range of activities such as lipase, and phospholipase activity was produced. In addition, these Cfp21 mutants could be considered as “Super enzymes” with enhanced TAG lipase and phospholipase activities since the lipase SA of the Cfp21-A1 was 15 fold higher than that of Cfp21-WT, and the PLA_1_ SA of Cfp21-A2 and Cfp21-A1A2 were 2.5 and 3.5 fold greater than that of the wild type Cut4, respectively. Moreover, apparent PLA_2_ SA and cytotoxicity effects of these three mutants were similar to those of Cut4. As cutinase-like proteins of *M. tuberculosis*, are very immunogenic proteins [22], [23], these Cfp21 mutants proteins should be evaluated for their potency to induce the host’s immune system. In addition, Cfp21 mutants could be useful tools for further studies on the physiological role of *M. tuberculosis* cutinase-like proteins in the lipid metabolism of bacteria.
